# Wyoming Valley Veterinary Medical Association1Reported for the Journal by Dr. J. H. Timberman.

**Published:** 1896-05

**Authors:** 


Secretary.

### WYOMING VALLEY VETERINARY MEDICAL ASSOCIATION.[1]

THE annual meeting was held at the Exchange Hotel, Wilkesbarre, Friday evening, April 24, 1896. The meeting was called to order at 8 P.M. by President Dr. Butterfield, of Montrose. The following members responded to roll-call: Drs. Butterfield, Mead, Church, Helmer, Hewitt, and Timberman, also Dr. Osborne, of Wilkesbarre, an applicant for membership. The minutes of the last meeting were read and approved.

The report of the Committee on Sanitary Science and Police brought out an interesting discussion on tuberculosis.

Dr. Church reported a case of "Fracture of Metatarsus." There was also a report by Dr. Butterfield of some interesting cases of "Malignant Catarrh" in cows.

Dr. Helmer then gave a talk on "Hog-cholera and Swine-plague." This was not written out in the form of a paper, but was a talk from notes and headings. The doctor handled the subject with much detail and in a manner which showed that he must have given the matter considerable thought and study. He defined the two diseases, and dwelt at length on the differential characteristics of each, with the best manner of handling an outbreak, means of disinfecting premises, isolating, etc. An animated and instructive discussion followed, which was participated in by all present.

This being the annual meeting election of officers followed. Dr. Butterfield was selected President; Dr. Mead, Vice-President; Dr. Church, Secretary and Treasurer. Board of Trustees or Censors, Drs. Helmer, Sturge, and Timberman.

On motion, the meeting adjourned to meet at Montrose on the last Friday evening in July.

Dr. Osborne was admitted to membership.

[1] Reported for the JOURNAL by Dr. J. H. Timberman.

